# Evaluation of DNA extraction from different canine lymphoma sample types for PARR analysis

**DOI:** 10.29374/2527-2179.bjvm007926

**Published:** 2026-10-02

**Authors:** Vinício Cascardo, Carla Beatriz Ventura Leite, Matheus de Souza Santana, Huarrisson Azevedo Santos, Thiago Souza Costa, Andresa Guimarães

**Affiliations:** 1 Programa de Residência em Medicina Veterinária – Patologia Clínica. Departamento de Medicina e Cirurgia Veterinária (DMCV), Instituto de Veterinária (IV), Universidade Federal Rural do Rio de Janeiro (UFRRJ). Seropédica, RJ, Brazil.; 2 Programa de Pós-Graduação em Medicina Veterinária, DMCV, IV, UFRRJ. Seropédica, RJ, Brazil.; 3 Programa de Pós-Graduação em Ciências Veterinárias, Departamento de Parasitologia Animal. IV, UFRRJ. Seropédica, RJ. Brazil.; 4 Departamento de Epidemiologia e Saúde Pública, IV, UFRRJ. Seropédica, RJ, Brazil.; 5 Hospital Veterinário, IV, UFRRJ. Seropédica, RJ, Brazil.; 6 DMCV, IV, UFRRJ. Seropédica, RJ, Brazil.

**Keywords:** diagnosis, dogs, polymerase chain reaction, oncology, diagnóstico, cães, reação em cadeia da polimerase, oncologia

## Abstract

This study evaluated the performance of DNA extraction using the phenol–chloroform method across different types of canine lymphoma samples, with the aim of applying it to PARR. A total of 48 samples previously diagnosed as lymphoma were analyzed, including lymph node aspirates, cytological slides, andparaffin-embedded tissue blocks. DNA concentration was measured by spectrophotometry, and DNA suitability was confirmed by conventional PCR targeting a lymphocyte-specific endogenous control. No statistically significant differences were observed among the groups regarding DNA concentration (p > 0.05), with the following pairwise comparisons: paraffin-embedded tissue versus aspirate (p = 0.6827), aspirate versus cytological slide (p = 0.2242), and paraffin-embedded tissue versus cytological slide (p = 0.1173). All samples showed successful amplification, and the method demonstrated consistent performance, enabling the use of a single extraction protocol applicable to all three sample types, thereby showing potential as an accessible and standardizable approach for preparing samples for the PARR assay.

## Resumo

Este estudo avaliou o desempenho da extração de DNA pelo método fenol-clorofórmio em diferentes tipos de amostras de linfoma canino, visando à sua aplicação na PARR. Foram analisadas 48 amostras previamente diagnosticadas como linfoma, incluindo aspirados de linfonodos, lâminas citológicas e blocos de tecido incluídos em parafina. A concentração de DNA foi determinada por espectrofotometria, e a adequação do DNA foi confirmada por PCR convencional direcionada a um controle endógeno específico de linfócitos. Não foram observadas diferenças estatisticamente significativas entre os grupos quanto à concentração de DNA (*p* > 0,05), considerando as seguintes comparações: tecido incluído em parafina versus aspirado (*p* = 0,6827), aspirado versus lâmina citológica (*p* = 0,2242) e tecido incluído em parafina versus lâmina citológica (*p* = 0,1173). Todas as amostras apresentaram amplificação bem-sucedida, e o método demonstrou desempenho consistente, possibilitando a utilização de um único protocolo de extração aplicável aos três tipos de amostras e demonstrando potencial como uma abordagem acessível e padronizável para o preparo de amostras destinadas ao ensaio de PARR.

## Introduction

Canine lymphoma is the most common hematopoietic neoplasm in dogs, and its diagnosis is primarily based on cytological, histopathological, and immunohistochemical evaluation, with additional support from molecular techniques (Gonçalves et al., 2021). PCR for Antigen Receptor Rearrangements (PARR) enables the assessment of lymphocyte clonality, allowing differentiation between reactive and neoplastic processes, as well as contributing to tumor phenotype characterization (Burnett et al., 2003). The efficiency and reproducibility of DNA extraction methods are critical for ensuring the reliability of this assay. Therefore, this study aimed to evaluate the performance of DNA extraction using the phenol–chloroform method across different types of clinical samples intended for PARR analysis.

## Materials and methods

A total of 48 samples previously diagnosed as lymphoma were analyzed, including 11 paraffin-embedded tissue blocks, 18 cytological slides, and 19 lymph node aspirates. Paraffin-embedded tissue blocks were subjected to prior deparaffinization using xylene followed by graded ethanol washes, cytological material was obtained by scraping the slides with a sterile scalpel blade and subsequently resuspended in PBS; lymph node aspirates were diluted in saline solution at the time of collection. The study was approved by the Animal Ethics Committee (CEUA; protocol no. 2060160424).

DNA extraction was performed based on the protocol described by Sambrook et al. (1989), with modifications. Samples were processed in 2 mL microtubes containing 350 μL of PBS, to which 500 μL of extraction buffer (10 mM Tris-HCl, pH 8.0; 10 mM EDTA, pH 8.0; 100 mM sodium chloride; and 2% SDS) and 20 μL of proteinase K (20 mg/mL) were added. After vortexing, the tubes were incubated at 56 °C for 1 h 30 min under agitation at 650 rpm. Subsequently, an equal volume of phenol (pH 8.0), corresponding to 870 μL, was added, followed by vortexing and centrifugation at 16,000 × g for 10 min. The supernatant was transferred to a new microtube containing 500 μL of chloroform, vortexed, and centrifuged again under the same conditions. The resulting supernatant was then transferred to a 1.5 mL microtube, and 50 μL of 3 M sodium acetate (pH 5.2) was added. A total of 500 μL of absolute ethanol was added, followed by vortexing and incubation at −20 °C for 1 h 30 min. DNA was recovered by centrifugation at 16,000 × g for 10 min, and the supernatant was discarded. The pellet was then washed with 500 μL of 70% ethanol and centrifuged again under the same conditions. After air-drying by inverting the tubes, the DNA was eluted in 70 μL of TE buffer.

DNA concentration was determined by spectrophotometry (NanoDrop 2000), and samples with concentrations exceeding 100 ng/µL were adjusted to this threshold. Statistical analyses were performed in BioEstat5.0. Following Lilliefors normality testing, DNA concentrations were compared using pairwise Mann-Whitney tests (5% significance), with effect sizes (r=Z /√N) calculated and interpreted according to Rosenthal (1991) and Cohen (1988). The DNA was then stored at −20 °C until PCR analysis. DNA viability was assessed by conventional PCR targeting the lymphocyte-specific IgM region, using primers described by Burnett et al. (2003). Amplification was performed according to the protocol of Nowosh et al. (2017), in reactions with a final volume of 24 µL containing 4 µL of DNA; the amplification products were analyzed on 2% agarose gels.

## Results

All 48 samples showed positive amplification in PCR targeting the endogenous control gene. The median DNA concentrations and interquartile ranges (Q1–Q3) were 183.40 ng/µL (38.35–536.75) for lymph node aspirates, 220.30 ng/µL (68.35–720.10) for paraffin-embedded tissue blocks, and 105.30 ng/µL (62.85–176.98) for cytological slides (Table 1). No statistically significant differences were observed among the groups: paraffin blocks versus aspirates (p = 0.6827), aspirates versus cytological slides (p = 0.2242), and paraffin blocks versus cytological slides (p = 0.1173). The comparison between aspirates and paraffin blocks showed a negligible effect (r = 0.08), while aspirates versus cytological slides (r = 0.20) and cytological slides versus paraffin blocks (r = 0.23) both showed small effect sizes (Cohen, 1988). The absorbance ratios 260/230 and 260/280 are indicators of DNA quality, with values above 1.8 considered acceptable. The mean 260/230 and 260/280 ratios were, respectively, 1.91 and 2.25 for aspirates, 1.88 and 2.16 for blocks, and 1.87 and 2.20 for slides, all within the recommended range (Koetsier & Cantor, 2019).

**Table 1:** Descriptive statistics of DNA concentration (ng/µL) across sample types.

	Aspirate	Paraffin block	Cytological slides
Sample size	19	11	18
Minimum	7.10	11.70	7.60
Maximum	1392.60	4241.30	911.60
Arithmetic mean	353.74	741.34	156.10
Median (Q1-Q3)	183.40 (38.35-536.75)	220.3 (68.35-720.1)	105.30 (62.85-176.98)
Standard deviation	424.25	1267.03	204.61

## Discussion

Positive amplification of the endogenous control demonstrates the efficiency of the protocol and confirms the suitability of all samples for PARR. Despite differences in sample type, phenol–chloroform extraction performed similarly across the three groups.

All 17 samples with concentrations below 100 ng/µL showed positive amplification, in contrast to Langner et al. (2014), who observed amplification only at higher DNA concentrations in some samples. In addition, Nowosh et al. (2017) reported the absence of detectable lymphoid DNA in 33% of cytological slides obtained from fine-needle aspirates, which was attributed to low yield or sample degradation.

Although DNA impairment in paraffin-embedded samples has been described (Keller & Moore, 2012), all samples subjected to prior deparaffinization showed positive amplification, in agreement with Gonçalves et al. (2021).

Compared to cytology slides, lymph node aspirates provide a larger amount of viable material for extraction but do not allow prior visualization of the collected material, increasing the risk of non-lymphoid sampling.

Cytological slides are a reliable source for molecular analyses (Killian et al., 2010), and all samples in this study yielded adequate DNA and positive amplification, supporting their use in PARR. This approach allows the integration of cytological and molecular findings, increasing diagnostic reliability (Keller & Moore, 2012), and enables the use of previously prepared slides, reducing the need for new procedures and facilitating diagnosis, prognostic assessment, and therapeutic decision-making.

A limitation of this study is that DNA suitability was evaluated only by concentration, purity, and endogenous control amplification. Although amplifiable DNA was obtained from all sample types, its performance in the complete PARR assay remains to be confirmed.

The phenol–chloroform extraction protocol showed potential as an efficient and accessible alternative, enabling a single extraction method applicable to all three sample types, thereby facilitating its standardization and incorporation into the routine diagnosis of canine lymphoma.

## Conclusion

The phenol–chloroform extraction technique showed consistent performance across all analyzed groups, with positive amplification of the endogenous control in all samples, indicating DNA integrity and supporting its potential use for the PARR assay.
